# Fullerenol changes metabolite responses differently depending on the iron status of cucumber plants

**DOI:** 10.1371/journal.pone.0251396

**Published:** 2021-05-17

**Authors:** Nikolai P. Bityutskii, Kirill L. Yakkonen, Roman Puzanskiy, Kseniia A. Lukina, Alexey L. Shavarda, Konstantin N. Semenov

**Affiliations:** 1 Department of Agricultural Chemistry, Saint Petersburg State University, Saint Petersburg, Russia; 2 Komarov Botanical Institute, Saint Petersburg, Russia; 3 Research Park, Centre for Molecular and Cell Technologies, Saint Petersburg State University, Saint Petersburg, Russia; 4 Department of General and Bioorganic Chemistry, First Pavlov State Medical University, Saint Petersburg, Russia; United Arab Emirates University, UNITED ARAB EMIRATES

## Abstract

The unique properties of carbon-based nanomaterials, including fullerenol, have attracted great interest in agricultural and environmental applications. Iron (Fe) is an essential micronutrient for major metabolic processes, for which a shortage causes chlorosis and reduces the yield of many crops cultivated worldwide. In the current study, the metabolic responses of *Cucumis sativus* (a Strategy I plant) to fullerenol treatments were investigated depending on the Fe status of plants. Cucumber plants were grown hydroponically, either with [+Fe^II^ (ferrous) and +Fe^III^ (ferric)] or in Fe-free (−Fe^II^ and −Fe^III^) nutrient solution, with (+F) or without (−F) a fullerenol supply. Iron species-dependent effects were observed in either Fe-fed or Fe-starved plants, with alteration of metabolites involved in the metabolism of carbohydrates, amino acids, organic acids, lipophilic compounds. Metabolic perturbations triggered by fullerenol in the Fe^III^-treated plants were in the opposite kind from those in the Fe^II^-treated plants. Whereas in the Fe^III^-fed plants, fullerenol activated the metabolisation of carbohydrates and amino acids, in the Fe^II^-fed plants, fullerenol activated the metabolisation of lipophilic compounds and repressed the metabolisation of carbohydrates and amino acids. In Fe^III^-deficient plants, fullerenol stimulated the metabolism of C_3_ carboxylates and lipophilic compounds while repressing the metabolism of amino acids, hexoses and dicarboxylates, while in Fe^II^-deficient plants, activations of the metabolism of amino acids and dicarboxylates and repression of sterol metabolism by fullerenol were observed. The results indicated that the valence state of Fe sources is of importance for re-programming metabolome responses in cucumber to fullerenol either in Fe-sufficient or Fe-deficient conditions. These investigations are significant for understanding fullerenol interactions and risk assessment in plants with different Fe statuses.

## Introduction

Iron (Fe) is an essential micronutrient for major metabolic processes in higher plants, including respiration and photosynthesis [1,2]. The physiological importance of Fe is based on its electronic structure, which is capable of the reversible changes in oxidation state: Fe^II^ (ferrous) and Fe^III^ (ferric) ions, of various electron-transfer reactions [3]. Although Fe is the most abundant element of the earth’s crust, in the rhizosphere, its concentrations are often limited due to the poor solubility of Fe oxides, especially Fe^III^ oxides, at the high pH values of alkaline and calcareous soils. Iron (Fe) shortage causes chlorosis of the youngest leaves and reduces the yield and quality of many crops; therefore, Fe homeostasis of plants is a highly active research area all over the world [4].

To cope with the Fe deficit, plants have developed two main strategies: Strategy I (dicotyledonous and non-grass monocotyledonous species) and Strategy II (grasses). The Strategy I mechanisms include (i) enhanced excretion of protons to the soil mediated by a plasma membrane-bound H^+^-ATPase, (ii) induction of a plasma membrane ferric chelate reductase (FC-R) enzyme and (iii) increased Fe^II^ transport [1,5]. The solubilisation of Fe compounds can also promote the release of organic compounds (phenols, flavins and organic acids) in some Strategy I species [6–8]. Roots of Strategy II plants (grasses) increase the secretion of phytosiderophores (PSs) and the induction of an Fe^III^-PS complex transport system [9].

Although plants have developed these strategies under Fe deficiency, chlorosis can affect both Strategy I and Strategy II plants [1]. To control Fe deficiency in crops, Fe fertilisers are used. However, the solubility of Fe determines its biological availability rather than its abundance, therefore the problem of Fe deficiency cannot be easily overcome by using Fe fertilisers [10]. Recent investigations have been shown that carbon-based engineered nanomaterials (ENMs) have the potential to increase crop productivity and protection [11–13]. Unique characteristics of ENMs, such as a higher surface area to volume ratio and reactivity compared with equivalent bulk materials, allow their application in different technologies [12,14]. Among carbon-based ENMs, fullerenols [C_60_(OH)_*x*_, *x* = 18–36] have attracted great interest [15]. Fullerenols are nano-sized water-soluble polyhydroxylated derivatives of fullerenes, perspective bioactive compounds with some mutual effects in plant science. Indeed, fullerenol increased the biomass, fruit yield and phytomedicine content in bitter melon (*Momordica charantiai*) [16]. Under stressful conditions (e.g. ultraviolet (UV)-B radiation, salt stress and the excess of salicylic acid), fullerenol application enhanced root elongation of barley (*Hordeum vulgare*) [17]. Seed priming with fullerenol regulated wheat growth and yield under salt-stress [18]. In addition, fullerenol stimulated hypocotyl elongation in *Arabidopsis* [19]. Foliar application of fullerenol alleviated drought impact in sugar beets and Fe chlorosis in cucumber [20,21]. Very recently, we found that fullerenol could protect cucumber plants against Fe deficiency through increased utilisation of root apoplastic Fe [22]. The beneficial effects were more pronounced in Fe^II^-starved plants, being higher in root apoplastic Fe, suggesting that fullerenol might facilitate Fe transport in plants, depending on their Fe status. Most of these studies observed plant changes at phenotypic and physiological levels, whereas the underlying mechanisms have been less elucidated.

Metabolomics is considered a powerful tool to understand complex environmental perturbations in biological systems, including nanoparticles [23]. Different environmental stresses have large impact on plant homeostasis and can cause serious disturbance in plant metabolism, affecting levels of metabolites in plant tissues. Drought stress affects changes in carbohydrate metabolism, accumulation of osmolytes and organic acids. In response to drought, primary metabolism switches to secondary metabolism to synthesize and accumulation of defense-related secondary metabolites, with phenolics groups mainly participate in drought resistance [24]. Compatible solutes, such as sucrose, proline and glycine betaine, accumulate in the salt-stressed plants to balance the osmotic pressure of the ions [25,26]. At the same time, seed priming with AgNPs enhanced the production of phenolic and flavonoids under salinity, thereby helping pearl millet plants to coupe with oxidative damage induced by these stress conditions [26]. Heavy metals can also participate in plant metabolism. Vanadium (V) compounds increased the production and exudation of secondary metabolites, such as isoflavones, in a *Trifolium pratense* suspension culture [27]. Flavonoids act as scavengers of free radicals or pro-oxidants to oppose adverse impact of heavy metals. Secondary metabolites (phenolic acids and flavonoids) play also an important role in insect–plant interactions. Cabbage exposed to fifty flea beetles showed a higher content of phenylalanine, a substrate for the synthesis of phenolic compounds and production of flavonoids [28]. However, insect-stressed cabbage did not have a significantly higher content of flavonoids, such as quercetin and kaempferol, compared to control [28]. At the same time, stressed by pest infestation cabbage leaves manifested an increase in ascorbic acid. It has been proposed that an increase in leaf ascorbate concentration can decrease the lifetime of ROS and change the balance of redox signaling pathways [29].

Other factors, such as different nutrient stresses can have negative effects on the biosynthesis of key metabolites which are critical for plant homeostasis [23]. To resist low nitrogen (N) stress, low-N-tolerant wild soybean decreased the synthesis of energy-consuming amino acids [30]. However, contents of soluble sugars, starch, carbohydrates, protein and amino acid in plants can be significantly enhanced by the treatment with the bio-organic fertilizers [31]. In magnesium (Mg) deficient soybean, most amino acids were accumulated in leaves, whereas most organic acids were decreased in roots [32]. In tea plants, some metabolites, such as organic acids and carbohydrates, were increased during zinc excess while other metabolites, such as carbohydrates and flavonoids, were decreased [33]. Molybdenum (Mo) deficiency significantly affected amino acids, sugars, organic acids and purine metabolites in *Arabidopsis thaliana* [34].

Several studies suggest that ENPs have a potential in activating plant metabolism; however, combination of both positive and negative effects of ENPs on plant growth and metabolism, including antioxidant machinery, has been documented [23,26,35]. The metabolic plant responses to ENPs exposure significantly varied with recipient species, on the one hand, and chemical composition, dose, particle size and aggregation level of the ENPs, on the other hand. Although extensive research has been carried out on ENPs, the metabolite responses of plants to fullerenol treatments remain largely unknown, including responses of plants with different Fe statuses. This metabolite approach enriches one’s knowledge for understand ENMs interactions and risk assessment in plants with different Fe status.

This work aimed to investigate how fullerenol changes metabolite responses of *Cucumis sativus* (a Strategy I plant) depending on its Fe status, with emphasis on fullerenol interactions with ferrous and ferric species used for Fe supply.

## Materials and methods

### Fullerenol synthesis and identification

Polyhydroxylated fullerene C_60_(OH)_22–24_ was synthesised and identified using previously developed methods, as described recently [21,36,37]. The identification of the obtained sample was performed utilising IR spectroscopy, elemental analysis, size distribution, and *ζ*-potentials study. The results of IR spectroscopy were as follows: 3418 cm^–1^ (*ν*O–H), 1597 cm^–1^ (*ν*C = C), 1370 cm^–1^ (*δ*_S_C–O–H) and 1060 cm^–1^ (*ν*C–O). Elemental analysis data: experimental—(C: 63.72%; H: 2.22%), calculated—(C: 63.83%; H: 2.13%). From the results of elemental analysis, a relative molar weight of 1128 g mol^–1^, corresponding to the C_60_(OH)_24_ formula, was applied in all further calculations. Size distribution revealed that the hydrodynamic diameters of fullerenol nanoparticles in the binary C_60_(OH)_22–24_–H_2_O system were 21 nm, and *ζ*-potentials values were −30 mV; therefore, indicating that even at low concentrations (*C* = 1 mg L^-1^), fullerenol forms associates in aqueous solution and the solutions are electrokinetically stable [21].

### Plant material and growth conditions

Cucumber (*Cucumis sativus* L.) is a Strategy I model plant for root responses to Fe deficiency, therefore it was be selected [1]. Cucumber seeds (*cv*. Phoenix) were obtained from the Vavilov Research Institute plant genetic resources (Saint Petersburg, Russia). Seeds (about 300 pieces) of cucumber were surface-sterilised and germinated between two sheets of filter paper moistened with distilled water at 28°C for four days in the dark. A detailed description of the experimental design has been reported recently [22]. Briefly, the seedlings were pre-incubated for 7 days in a complete nutrient solution containing (mM): 0.7 K_2_SO_4_, 0.1 KCl, 2.0 Ca(NO_3_)_2_, 0.5 MgSO_4_, 0.1 KH_2_PO_4_, and (μM): 1.0 MnSO_4_, 1.0 ZnSO_4_, 0.5 CuSO_4_, 0.01 (NH_4_)_6_Mo_7_O_24_ and 10 H_3_BO_3_. Iron (Fe) was added in form of Fe^II^–EDTA and Fe^III^–EDTA at 10 μM. Then, the pre-cultured seedlings were transferred to 1-L plastic pots (three plants per pot) and exposed to the same nutrition solution, either with (+Fe^III^ and +Fe^II^) or in Fe-free (−Fe^III^ and −Fe^II^) nutrient solution, with (+F) or without (−F) a fullerenol supply for 10 days. Fullerenol was added at final concentrations of 1 (F1) and 2 (F2) mg L^-1^, respectively, freshly prepared in distilled water. The control plants were the +Fe^III^-plants because ferric iron is the most abundant form of Fe in soils. If necessary, we used other treatments as control plants: the +Fe^II^-plants in case of study metabolic responses of the–Fe^II^-plants. The pH was adjusted to 6.0. The nutrient solutions were completely renewed every two days. Plants were grown with a photoperiod 16/8 h, a photon flux density of 200 μmol m^-2^ s^-1^ at plant height, temperature (light/dark) 24 ± 2°C/20 ± 2°C.

### Gas chromatography-mass spectrometry (GC-MS) of the leaf tissues

Metabolites were extracted, and subsequently, the extracts were derivatised and analysed using GC-MS, as described previously [38]. To prepare extract for single analysis 0.1 g of fresh leaf sample was used. Five replicate pots were used per treatment. Metabolites were analysed using an Agilent 7820 gas chromatograph equipped with a 5975 mass selective detector (Agilent Technologies, USA). The metabolites were normalised using tricosane (Sigma-Aldrich, USA) as an internal standard and the dry weight.

### Construction of the metabolic network map and statistical analysis

GC-MS data analysis was performed using PARADISe software [39] in association with NIST MS Search (National Institute of Standards and Technology (NIST, USA). For additional metabolite identification, the AMDIS (Automated Mass Spectral Deconvolution and Identification System, NIST, USA) were used with mass-spectra libraries: NIST2010, Golm Metabolome Database (GMD) and in-house library of the Resource Centre “The Development of Molecular and Cell Technologies” of the St. Petersburg University. Mass-spectra were considered reliably identified if they coincided with the library record with a match factor of at least 80 (800 for NIST search). In addition, retention indices (RI) determined by calibration with standard alkanes were used for identification. A table of identified metabolites with matching factors and RI are in the supplementary material (S1 Table).

Data were processed in the environment of the R language 3.6.4 [40]. Arbitrary contents were calculated by normalisation peak areas by internal standard (tricosane) peak area. For quantitative interpretation, the data were normalised by the median of the sample. The data were log_2_-transformed and standardised. Outlying values were excluded from the analysis with Dixon’s test. When a metabolite was not detected but was present in other replicated samples (at least half replicates), it was postulated as technical error and missing values were imputed. Missing data imputation was performed by KNN (k-nearest neighbours) with the *impute* R package [41]. Finale table of arbitrary metabolite content attached to supplementary (S2 Table).

A heat map was made by the package *ComplexHeatmap* [42]. PCA (principal component analysis) was realised with *pcaMethods* [43]. OPLS-DA was made with *ropls* [44]. Variables with VIP (variable importance in the projection) > 1 which played significant roles in the classification, were selected for further analysis.

For the enrichment analysis, the *fgsea* package was used [45]. As a ranking statistics factor, loadings of the predictive components from OPLS-DA models were used. Pathways associated with our metabolite set were extracted from KEGG with the *KEGGREST* package [46]. Revealed lists of metabolites for pathways were manually curated and metabolites identified just up to class were included in the pathways related to these groups. Pathways were mapped with the software environment of Cytoscape, using «organic layout» [47]. In graph nodes were assigned to KEGG pathways, edges represent common metabolites. Metabolites (nodes) were mapped by significant (p<0.05) Pearson correlation coefficients (edges) using “edge-weighted spring embedded layout”.

## Results

### General overview of the metabolite profile

**Fig 1:**
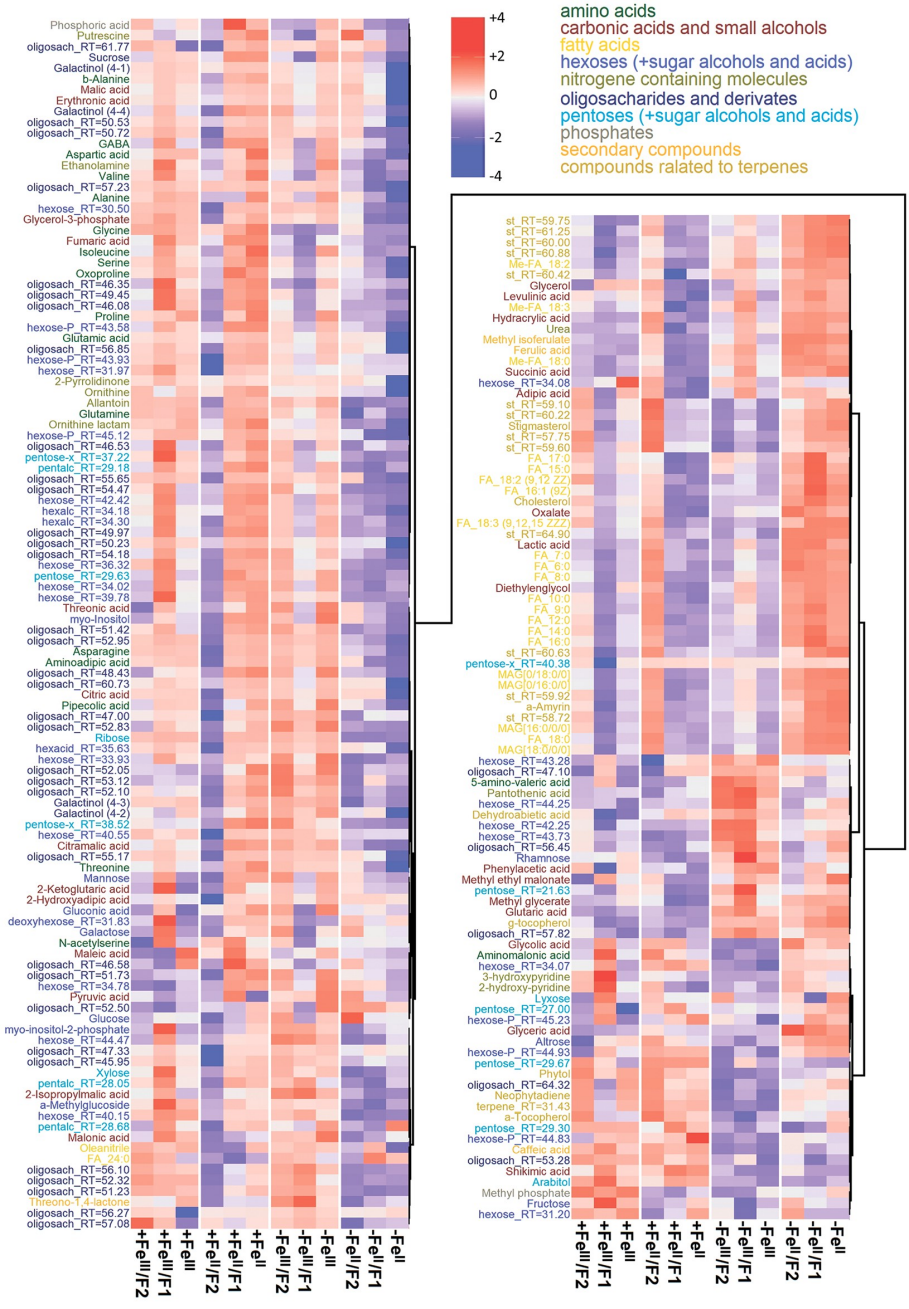
Heat map of average arbitrary content of identified (at least up to class) metabolites in the cucumber leaves grown hydroponically in a nutrient solution, either with (+Fe^II^ and +Fe^III^) or in Fe-free (−Fe^II^ and −Fe^III^) nutrient solution, with or without the supply of 0 (F0), 1 (F1) and 2 (F2) mg L^-1^ fullerenol for 10 days. Data are normalised to the median of observation, log-transformed and standardised. The map is combined with the dendrogram of hierarchical clustering using the Spearman distance (1 − r), clusters are agglomerated according to the Ward method. Keys: “+”—presence, “-”—absence, “F”—fullerenol in media, “F1”—1 mg L^-1^, “F2”—2 mg L^-^_1_, in metabolite names: “RT”—retention time, “MAG”—monoacylglycerol, in square brackets—fatty acids at corresponding positions, “-P”—phosphate, “hexalc” and “pentalc”—sugar alcohols of hexoses and pentoses, analogously “hexacid” and “pentacid”—sugar acids, “olygosach”—oligosaccharides or their derivatives.

The metabolite profiles of cucumber leaves were analysed. Obtained profiles included about 400 metabolites, 110 of which were identified exactly to compounds, and 90 spectra were assigned to a specific chemical class. The remaining mass spectra were not identified. Most abundant among the identified metabolites were sugars and their derivatives (95), including oligosaccharides: pentoses, hexoses, sugar alcohols, and sugar acids. Furthermore, amino acids (19, among them 12 ones are proteinogenic), carboxylic acids and energy metabolism intermediates (25), fatty acids and their derivatives (22), as well as nitrogenous bases, sterols and other compounds were detected. The results were visualised as a heat map combined with a dendrogram of hierarchical clustering with Spearman’s distance (1 − *r*) (Fig 1).

**Fig 2:**
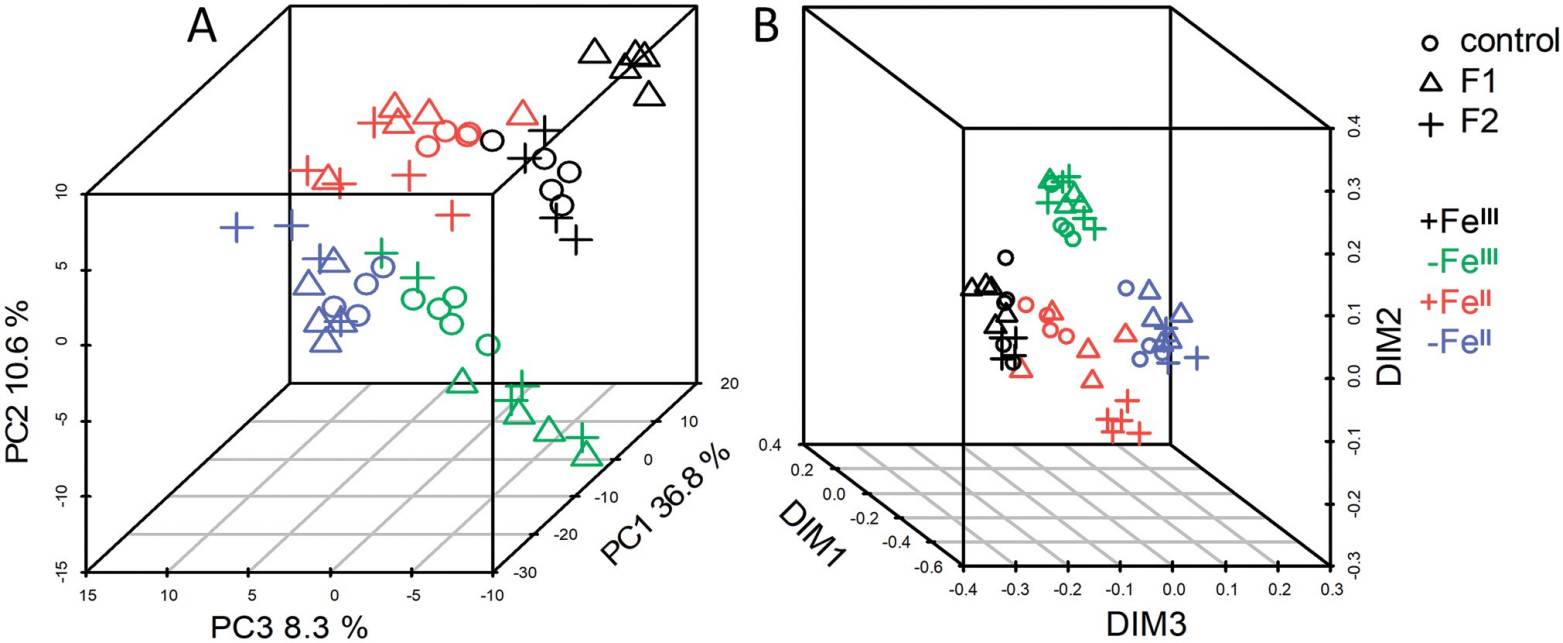
Unsupervised dimension reduction. Representing metabolite profiles of cucumber leaves grown hydroponically in a nutrient solution, either with (+Fe^II^ and +Fe^III^) or in Fe-free (−Fe^II^ and −Fe^III^) nutrient solution, with or without the supply of 0 (F0), 1 (F1) and 2 (F2) mg L^-1^ fullerenol for 10 days in low dimensional space. Data are normalised to the median of observation, log-transformed and standardised. A—PCA score plot, %—the variance associated with the PC. B—representation of metabolite profiles in space obtained using MDS (multidimensional scaling) using Spearman distance (1 − r).

To resolve the similarity of the obtained metabolite profiles, they were represented in the lower dimensional space of the scores derived from the PCA. The first three PCs represented 37, 11 and 8% of the variance, respectively (Fig 2A). As can be seen from the figure, profiles are clearly grouped in the space of these three components accordingly to the Fe-status of the plants. Using the multidimensional scaling (MDS) with the 1 − *r* value as a distance measure (*r* is the Spearman correlation coefficient) gave an even more distinct clustering of metabolite profiles depending on the Fe status of plants (Fig 2B). Thus, the Fe status of plants (+/−Fe; Fe^II^/Fe^III^) was a leading factor that controlled the metabolome (i.e. carbon redistribution between metabolite pools) of cucumber leaves under the experimental conditions.

### Metabolic responses in Fe-sufficient plants to Fe species

**Fig 3:**
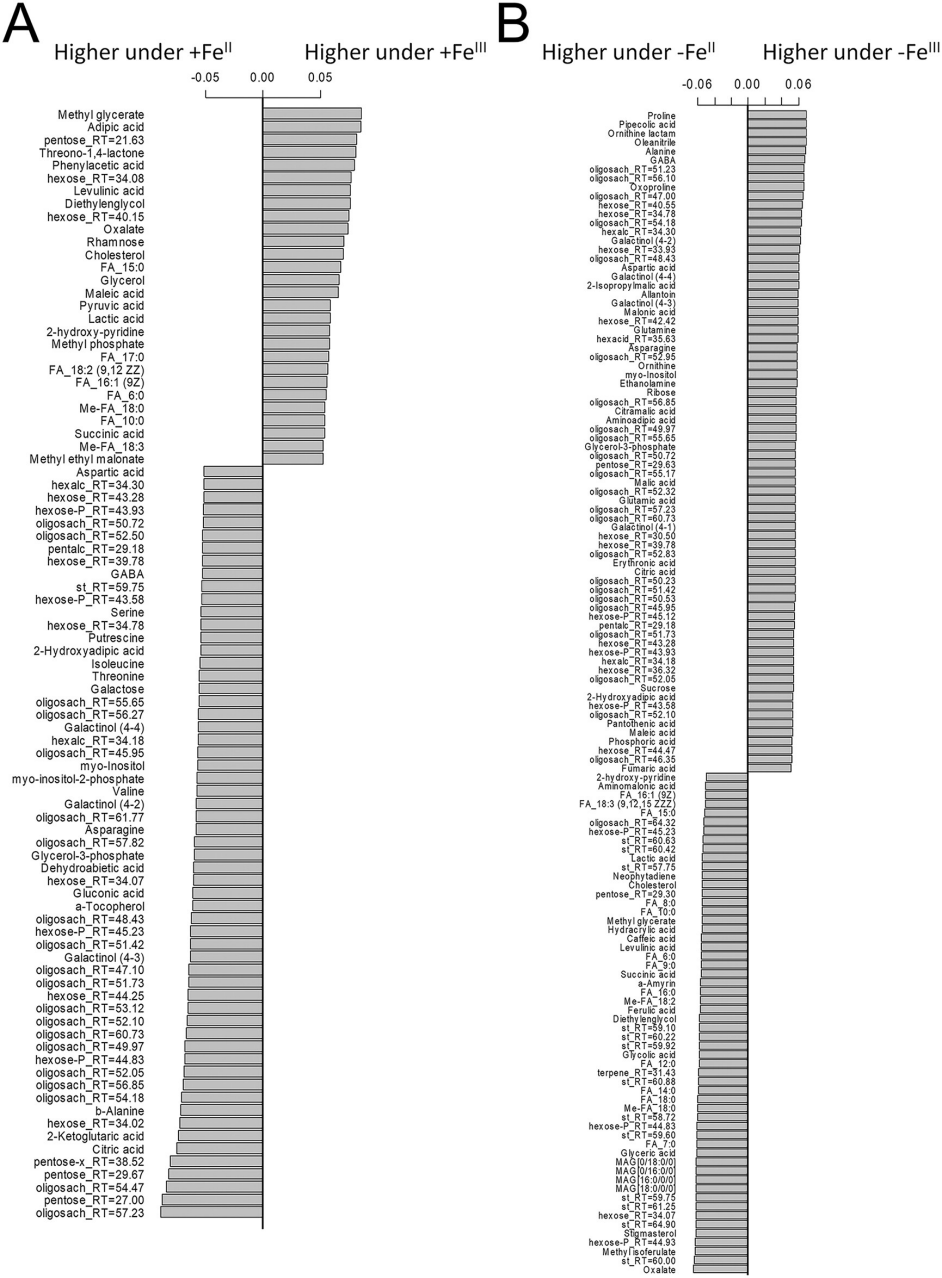
Selection of features discriminating cucumber plants grown hydroponically in a nutrient solution, either with (+Fe^II^ and +Fe^III^) or in Fe-free (−Fe^II^ and −Fe^III^) nutrient solution, with or without the supply of 0 (F0), 1 (F1) and 2 (F2) mg L^-1^ fullerenol for 10 days. Barplots of loadings of predictive component (VIP > 1) derived from OPLS-DA comparing of the metabolome of cucumber grown in a nutrient solution with Fe^II^ or Fe^III^: A—Fe-sufficient plants, B—Fe-deficient plants.

To select metabolites related to the plant Fe species, the metabolite profiles were analysed using the OPLS-DA method. The revealed OPLS-DA model included a predictive and one orthogonal component, R^2^X = 0.68, R^2^Y = 0.99 (*p* = 0.02), Q^2^Y = 0.82 (*p* = 0.05). A predictive component representing the effect of the action of the experimental factor explained 29% of the variance. Analysis of the factor loadings of the predictive component (VIP > 1) revealed numerous differences in the metabolism between the +Fe^III^ and +Fe^II^ plants (Fig 3A). In the +Fe^II^ plants, among metabolites with higher contents various sugars and amino acids prevailed, such as asparagine, valine, b-alanine, threonine, isoleucine, serine, GABA and aspartate. Additionally, the +Fe^II^ plants demonstrated the highest contents of citrate and 2-ketoglutarate. By contrast, the leaves of +Fe^III^ plants were more enriched with fatty acids, as well as some carboxylic acids, including oxalate, pyruvate, lactate, succinate and malate (Fig 3A).

### Effects of fullerenol on Fe-sufficient plants

**Fig 4:**
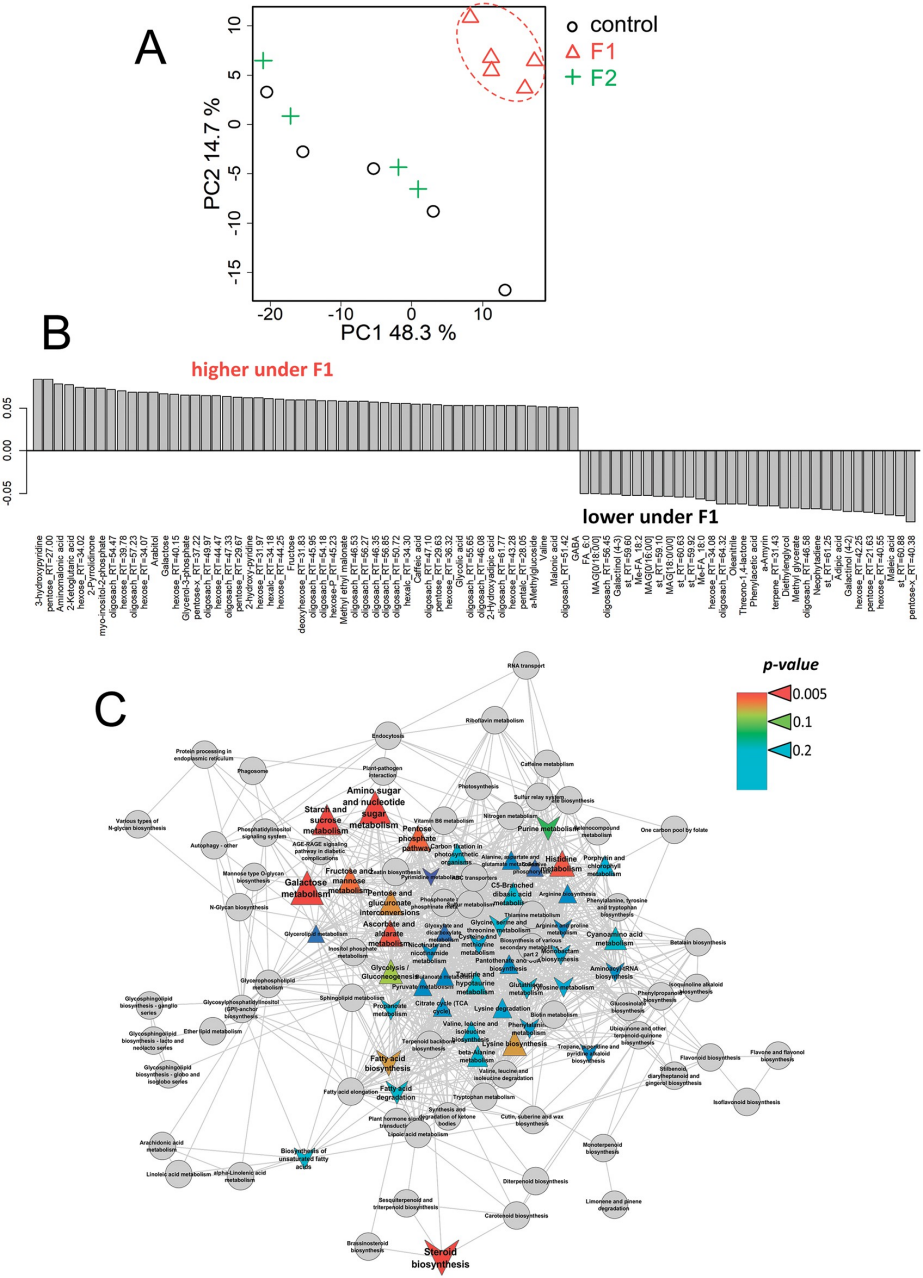
Fullerenol effects on leaf metabolome of the Fe^III^-sufficient cucumber plants grown hydroponically in a nutrient solution with or without the supply of 1 (F1) mg L^-1^ fullerenol for 10 days. A—PCA score plot, B—Bar plots of loadings of predictive component (VIP > 1) derived from OPLS-DA, C—Enrichment analysis. Colours represent *p-*value, shapes: Δ—activation induced by F1, v—repression by Fq, sizes: The power of effect represented as |NES|.

The principal component analysis revealed two PCs, reflecting 48 and 15% of the variance (Fig 4A). In the space of these PCs, metabolite profiles of +Fe^III^ plants grown in a medium with fullerenol (F1) clearly differed from control plants grown without fullerenol. Thus, fullerenol treatment affected the metabolic state of plants supplied with Fe^III^. The OPLS-DA model included a predictive and one orthogonal component: R^2^X = 0.69, R^2^Y = 0.99 (p = 0.01), Q^2^Y = 0.91 (p = 0.01). The predictive component represented 35% of the variance. The bar plot of loadings of the predictive component with VIP > 1 is shown in Fig 4B. According to these data, fullerenol mostly stimulated the accumulation of sugars (mainly oligosaccharides) in leaves of the +Fe^III^ plants, as well as of some amino acids (valine, GABA, aminomalonate) and carboxylates (glycolate, malonate). At the same time, the content of certain fatty acids, acylglycerols and sterols, as well as sugars (mainly monosaccharides) and carboxylates (maleate) decreased. Enrichment analysis showed that the effects of fullerenol (F1) were associated with the activation of carbohydrate metabolism, including the pentose phosphate pathway and starch metabolism (Fig 4C). In addition, fullerenol induced an increase in the pools of the intermediates in histidine metabolism. Notably, there was a stimulation of ascorbate metabolism by the fullerenol.

**Fig 5:**
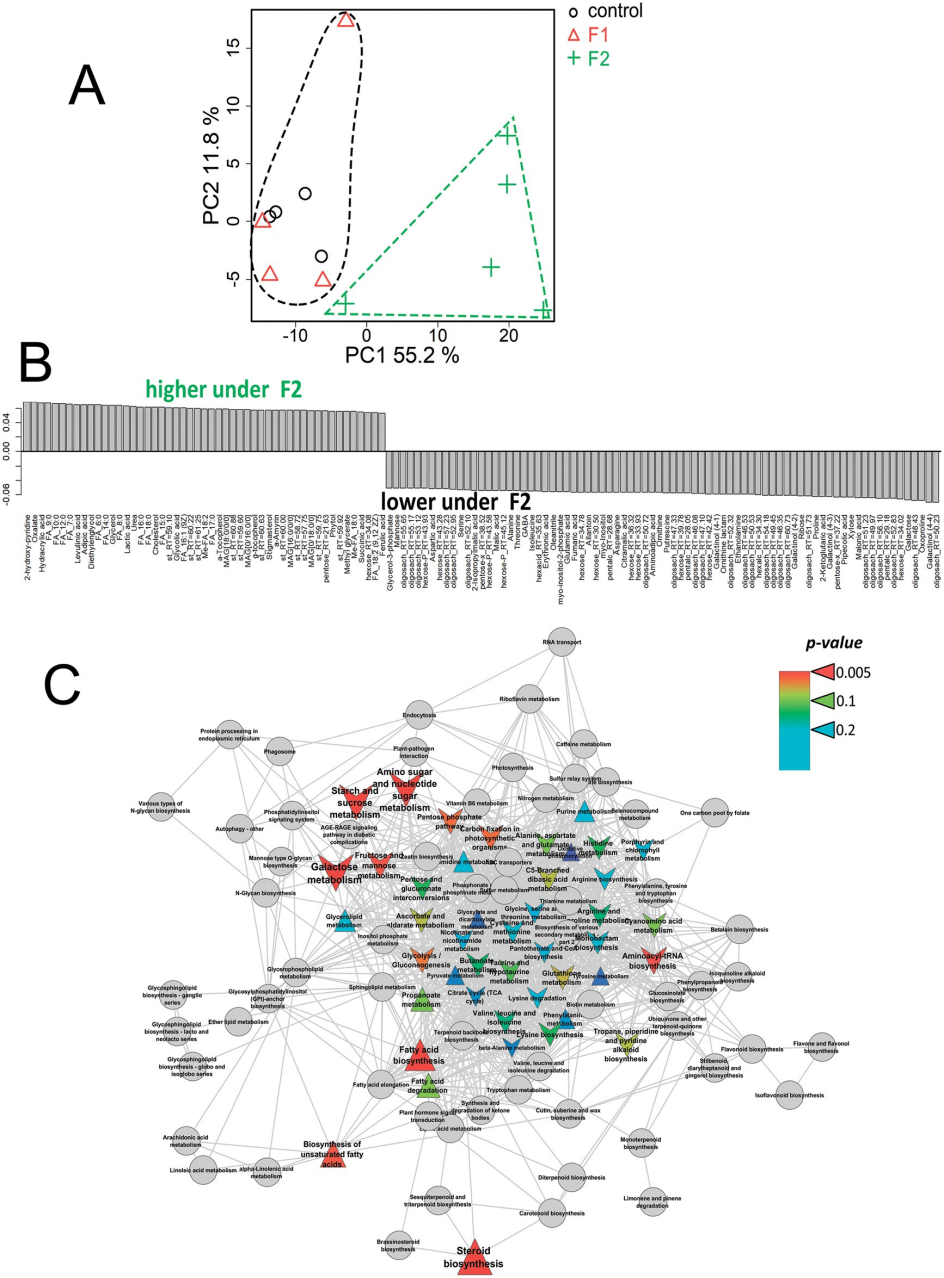
Fullerenol effects on leaf metabolome of the Fe^II^-sufficient cucumber plants grown hydroponically in a nutrient solution with or without the supply of 2 (F2) mg L^-1^ fullerenol for 10 days. A—PCA score plot, B—Bar plots of loadings of predictive component (VIP > 1) derived from OPLS-DA, C—Enrichment analysis. Colours represent *p-*values, shapes: Δ—activation induced by F1, v—repression by Fq, sizes: The power of effect represented as |NES|.

Only fullerenol at the highest dose (F2) significantly changed the metabolome of the +Fe^II^ plants (Fig 5A). OPLS-DA models, including the predictive and orthogonal components, were calculated: R^2^X = 0.70, R^2^Y = 0.98 (p = 0.02); Q^2^Y = 0.77 (p = 0.02). There was 50% of the variation that was related to the predictive component. An analysis of the factor loadings of the predictive component with VIP > 1 showed that fullerenol stimulated the accumulation of many fatty acids, acylglycerols and sterols in the +Fe^II^ plants (Fig 5B). Many carboxylates also accumulated in plants grown with fullerenol: oxalate, glycolate, succinate, methylglycerate, hydroxypropionate, levulinate and adipate, among others. By contrast, fullerenol decreased the accumulation of sugars (oligosaccharides, monosaccharides and others), amino acids and nitrogen-containing compounds. Some carboxylates (malonate, fumarate, malate and 2-ketoglutarate) were also found in lower quantities in fullerenol treated plants than in the non-treated +Fe^II^ plants. Enrichment analysis showed the fullerenol induced activation of metabolism of lipophilic compounds, sterols and fatty acids in the +Fe^II^ plants (Fig 5B). At the same time, a dramatic decrease in the intensity of carbohydrate metabolism, including starch and carbon fixation was observed. In addition, there was a repression of the exchange of amino acids.

### Metabolic responses in Fe^III^-deficient plants

**Fig 6:**
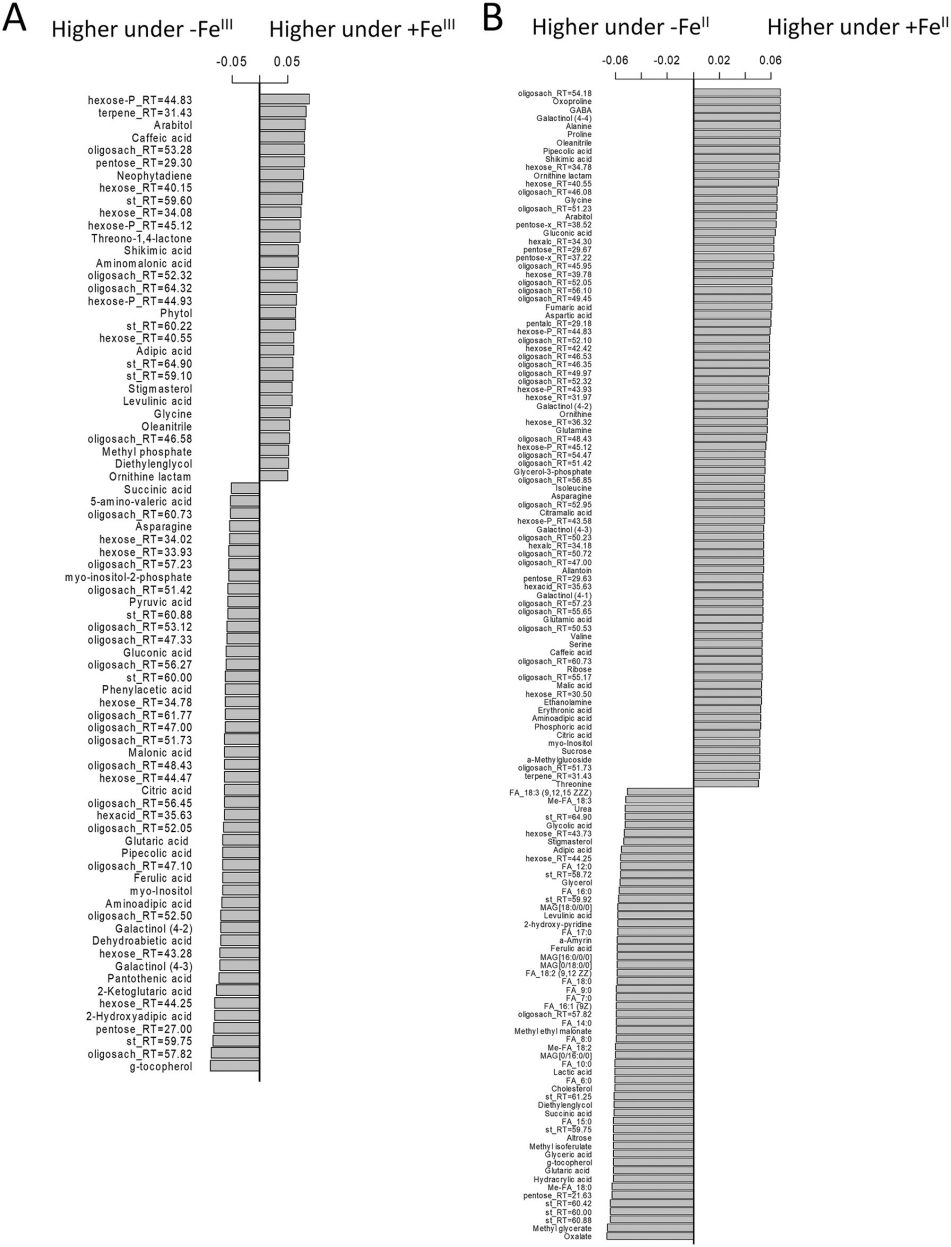
Selection of features discriminating leaf metabolome of the +Fe and −Fe cucumber plants grown hydroponically in a nutrient solution, either with (+Fe^II^ and +Fe^III^) or in Fe-free (−Fe^II^ and −Fe^III^) nutrient solution for 10 days. Bar plots of loadings of predictive component (VIP > 1) derived from OPLS-DA comparing of metabolomes of cucumber grown with different Fe species: A—Fe^III^ plants, B—Fe^II^ plants.

The OPLS-DA model for comparing the +Fe^III^ with–Fe^III^ plants included predictive and orthogonal components: R^2^X = 0.66, R^2^Y = 0.99 (*p* = 0.02), Q^2^Y = 0.90 (*p* = 0.02). The percent of variation associated with the predictive component was 29%. Thus, the metabolite profiles of the +Fe^III^ and–Fe^III^ plants were significantly different. Remove of Fe^III^ from a nutrient solution increased the leaf contents of various unidentified oligosaccharides and organic acids, including 2-hydroxyadipate, 2-ketoglutarate, glutarate, citrate, malonate, pyruvate, succinate and asparagine (Fig 6A). A decrease in the content caused by Fe^III^ deficiency was detected for some unidentified oligosaccharides, sterols (stigmasterol), terpenes (phytol) and secondary compounds (caffeic acid).

### Metabolic responses in Fe^II^-deficient plants

The OPLS-DA model for metabolomes of the +Fe^II^ and–Fe^II^ plants consists of a predictive and orthogonal component, R^2^X = 0.79, R^2^Y = 0.99 (*p* = 0.04), Q^2^Y = 0.90 (*p* = 0.04). In this case, 56% of the variance was explained by the predictive component. A lack of Fe^II^ induced an increase in the level of carboxylates (oxalate, hydracrylate, glycerate, succinate and lactate, among others) (Fig 6B). In addition, when Fe^II^ was removed from a nutrient solution, the leaf content of many amino acids decreased: proline, alanine, aspartate, oxoproline, GABA, glycine, serine, valine, glutamine, etc. In addition, some carboxylates, such as citrate, malate and fumarate, were downregulated. The level of many oligosaccharides, including sucrose, also decreased. The metabolic responses to Fe-starvation of plants pre-treated with different Fe sources were quite different. As was calculated, 64% of metabolites had loadings with opposite signs, indicating opposite directions of metabolic changes in response to different Fe sources. Thus, it can be concluded, that Fe valence plays a critical role in the following reaction on Fe starvation.

### Comparison of metabolic responses in the Fe^III^-deficient and Fe^II^-deficient plants

OPLS-DA of differences in metabolic profiles between the–Fe^II^ and–Fe^III^ plants revealed the following patterns. As in the previous case, the OPLS-DA model included a predictive and orthogonal component, R^2^X = 0.74, R^2^Y = 0.99 (*p* = 0.02), Q^2^Y = 0.81 (*p* = 0.02). At the same time, 50% of the variation was represented by a predictive component, which indicates an increase in the differences between plants supplied with various sources of Fe after their removal from the nutrient solution. The–Fe^III^ plants were characterised by an increased content of various sugars (Fig 3B), including sucrose. Relatively larger pools of many amino acids were also observed: proline, alanine, GABA, oxoproline, aspartate and others. Among carboxylic acids, isopropyl malate, malonate, citramalate, malate, citrate, maleate, fumarate exhibited higher contents in the–Fe^III^ plants. Plants lacking Fe^II^ showed a higher level of lipophilic compounds such as fatty acids, acylglycerols and sterols. In addition, carboxylates, such as oxalate, glycolate, succinate, lactate, etc., were upregulated in leaves of the–Fe^II^ plants (Fig 3B).

**Fig 7:**
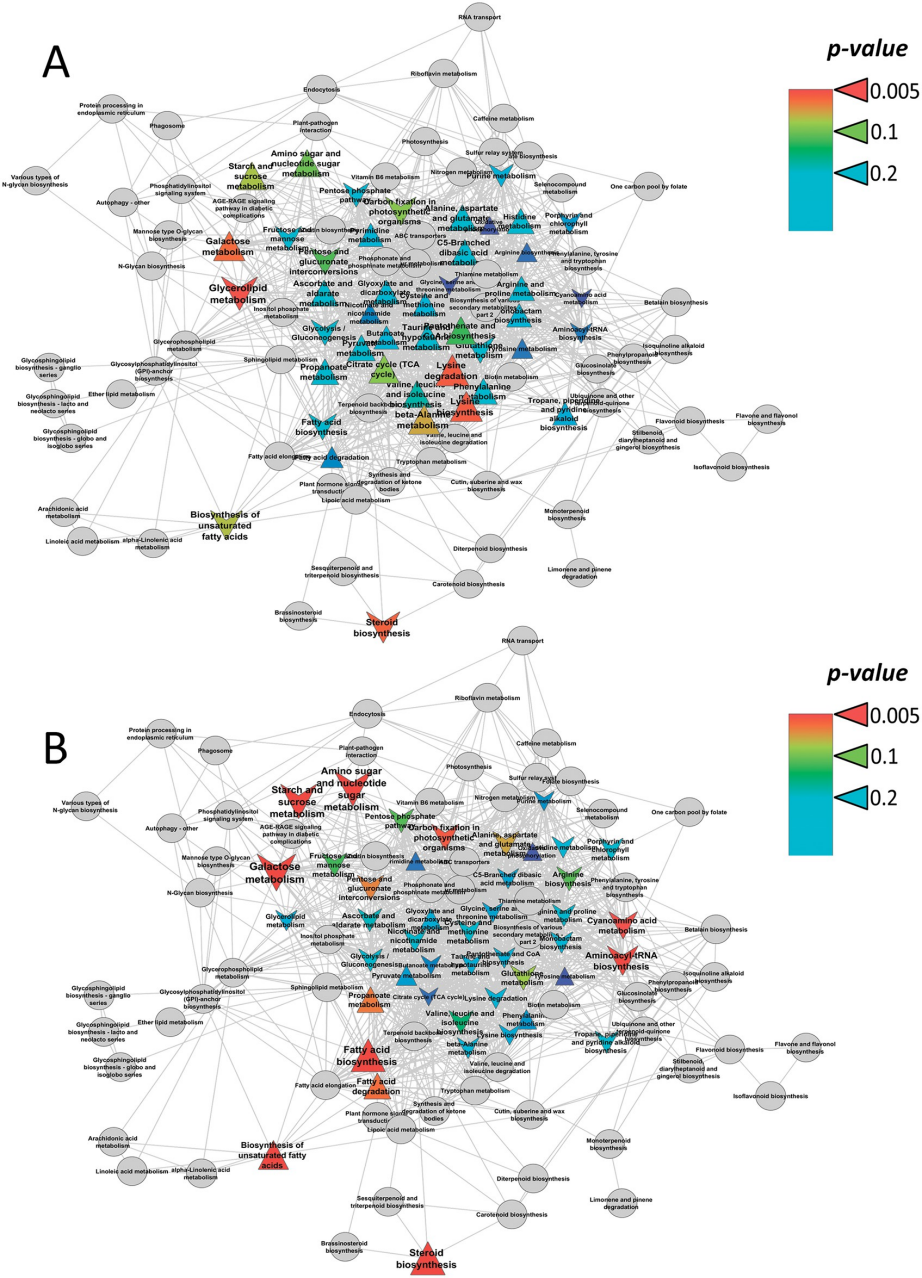
Enrichment analysis of the Fe deficiency effects on the leaf metabolism of cucumber grown hydroponically in a nutrient solution, either with/without +Fe^III^ (A) or with/without Fe^II^ (B). The graph represents a map of the biochemical pathways (nodes) connected by edges if they share common metabolites. The proximity of nodes determined from the number of common neighbours. Colours represent *p*-values, shape: Δ—activation under Fe starvation and v—repression under Fe starvation, sizes: The power of effect represented as |NES|.

Metabolic rearrangements during the transition of plants to Fe deficiency significantly varied in dependence on Fe species (Fig 7A). The exclusion of Fe^III^ from a nutrient solution caused, on the one hand, activation of pathways associated with the metabolism of aliphatic amino acids, and, on the other hand, repression of lipid metabolism. In contrast, Fe^II^ deficiency activated various pathways associated with lipid metabolism, including fatty acids and sterols (Fig 7B). The stimulation effect is also noted for the exchange of C_3_ metabolites. Metabolic repression was observed for amino acids, as well as sugars, including starch metabolism, hexoses and pentoses. In addition, carbon fixation was negatively affected by Fe^II^ deficiency.

To evaluate the degree of influence of Fe starvation on the difference between plants grown with Fe^III^ and Fe^II^, correlation analysis of loadings derived from corresponding OPLS-DA models was performed. It turned out the Spearman’s correlation was negative (r = −0.41, *p* <10^−16^). This indicates that the picture of the effect of Fe valence on the metabolism is reversed after Fe removal.

### Correlations of metabolite-metabolite in plants under different Fe conditions

**Fig 8:**
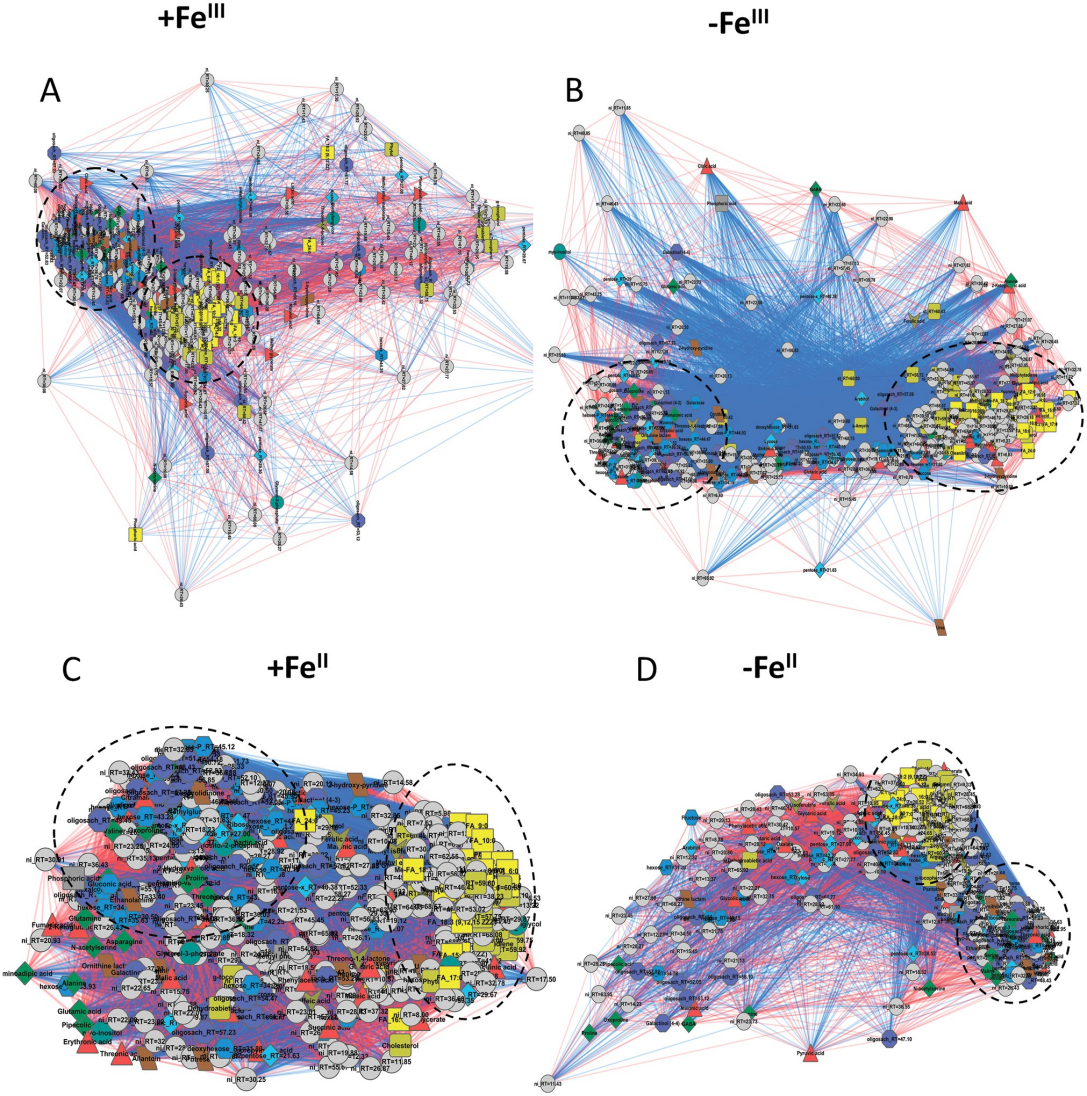
Mapping of leaf metabolites by correlation of the content levels in cucumber plants grown hydroponically in a nutrient solution, either with (+Fe^II^ and +Fe^III^) or in Fe-free (−Fe^II^ and −Fe^III^) nutrient solution for 10 days. The nodes are metabolites; the shapes and colours reflect the chemical nature of the compound. Edges are strong correlations (r > 0.7).

Correlation of metabolites allows evaluating metabolic connections and unveiling co-regulated blocks, which can change under external conditions. The results of the mapping of metabolites by correlations of their concentrations are presented in Fig 8. A common feature for all graphs is the presence of two clusters: “lipophilic” and “carbohydrate-amino acid”. According to the distribution of the correlation levels, it can be concluded that in the case of the–Fe^III^ plants (S1 File), the number of strong correlations declined in comparison to the +Fe^III^ plants (Fig 8). Probably, because of these changes, the structure of the network rearranged, in particular, clusters diverged. The transfer of plants to Fe^II^ starvation was accompanied by opposite changes. An increase in the number of strong correlations observed, which led to local consolidation of network and sharper clusters. Thus, the Fe^II^ deficiency caused apparently larger scale metabolic rearrangements in comparison with the Fe^III^ deficiency. They were realised in numerous coordinated changes in the metabolite contents, being reflected in a strengthening of correlation bonds (Fig 8, S1 File).

### Effects of fullerenol on Fe^III^-deficient plants

**Fig 9:**
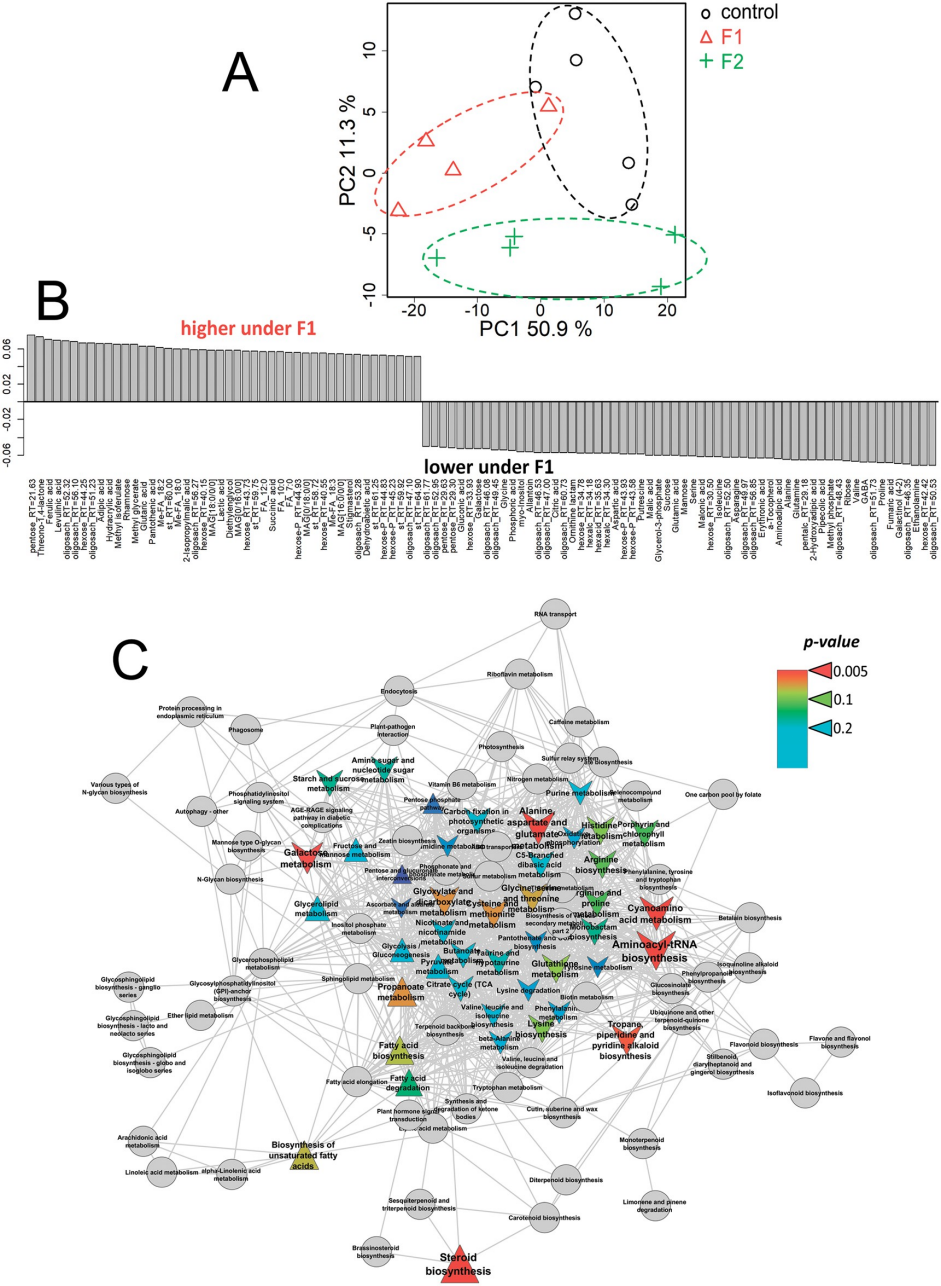
Fullerenols effects on leaf metabolome of the Fe^III^-deficient cucumber plants grown hydroponically in a nutrient solution with or without the supply of 1 (F1) mg L^-1^ fullerenol for 10 days. A—PCA score plot, B—Bar plots of loadings of predictive component (VIP > 1) derived from OPLS-DA, C—Enrichment analysis. Colours represent *p-*values, shapes: Δ—activation induced by F1 and v—repression by Fq, sizes—the power of effect represented as |NES|.

According to PCA, under conditions of Fe^III^ deficiency, fullerenol at low concentration (F1) had the greatest effect on the metabolic profiles of cucumber plants (Fig 9A, S1 File). The OPLS-DA model included predictive and one orthogonal components, R^2^X = 0.64, R^2^Y = 0.96 (p < 0.01), Q^2^Y = 0.73 (p < 0.01). The predictive component was associated with 42% of the variance, which is comparable to values obtained for the +Fe^III^ plants. The OPLS-DA model for control plants and plants treated with fullerenol at higher concentrations (F2) was insignificant. In contrast to the +Fe^III^ plants, treatment of the–Fe^III^ plants with fullerenol (F1) increased the content of lipophilic compounds such as fatty acids, acylglycerols and sterols (Fig 9B). In addition, fullerenol at F1 stimulated the accumulation of many carboxylates in the leaves: adipate, ferulate, levulinate, glutarate, succinate and some others. On the other hand, the levels of fumarate, malonate, malate, citrate and some other carboxylates decreased. Amino acids and other nitrogen-containing compounds also decreased in response to fullerenol (F1) treatments, whilst sugars showed mixed trends. Enrichment analysis showed fullerenol induced inactivation in the Fe^III^-deficient plants of various biochemical pathways, including metabolisation of amino acids, hexoses, dicarboxylates (Fig 9C). On the other hand, fullerenol stimulated a forming of intermediate pools in the metabolisation of C_3_ carboxylates and lipophilic compounds.

### Effects of fullerenol on Fe^II^-deficient plants

**Fig 10:**
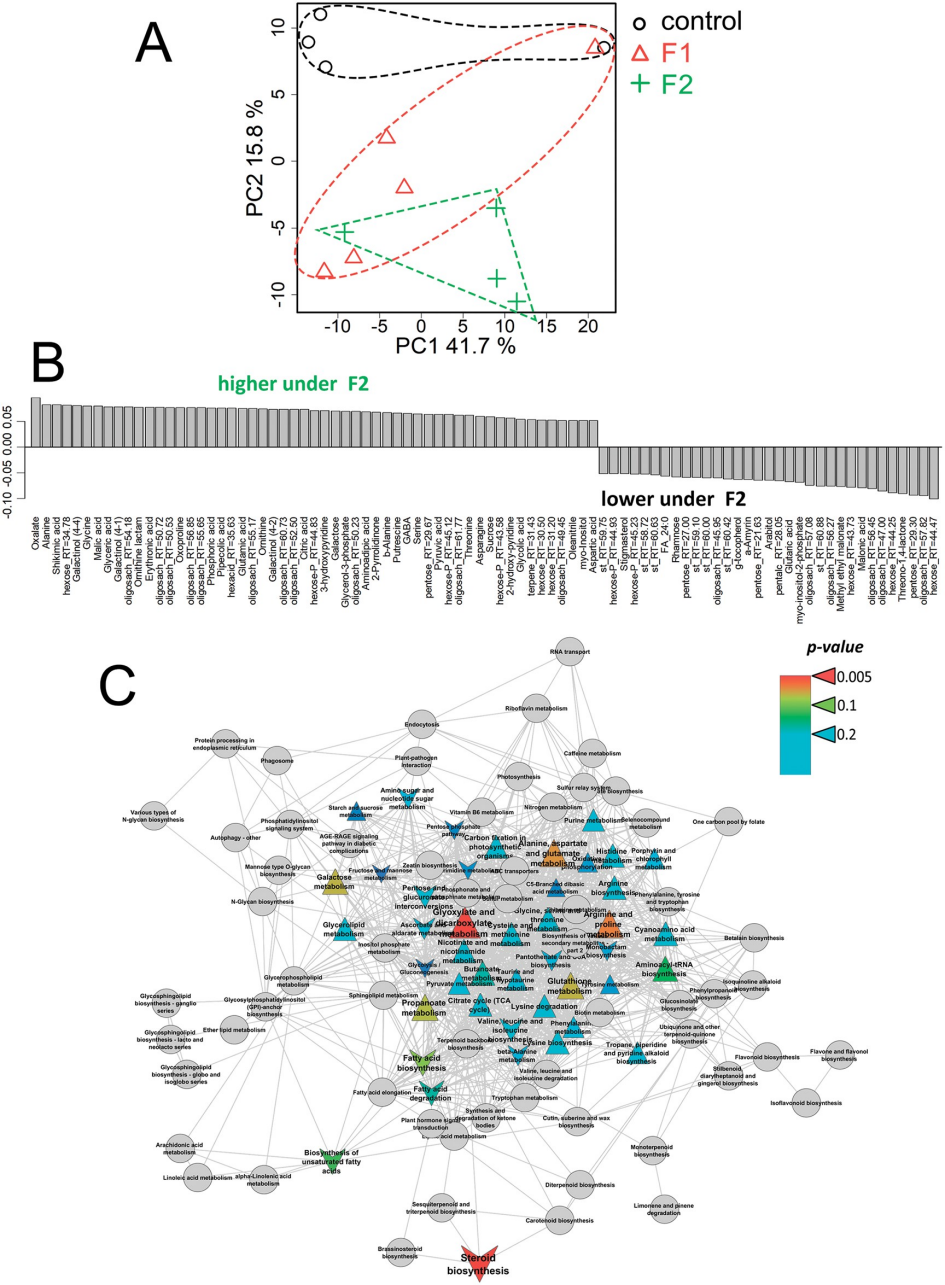
Fullerenols effects on leaf metabolome of the Fe^II^-deficient cucumber plants grown hydroponically in a nutrient solution with or without the supply of 2 (F2) mg L^-1^ fullerenol for 10 days. A—PCA score plot, B—Bar plots of loadings of predictive component (VIP > 1) derived from OPLS-DA, C—Enrichment analysis. Colours represent *p-*values, shape: Δ—activation induced by F1 and v—repression by Fq, sizes: The power of effect represented as |NES|.

Under Fe^II^ deficiency, metabolite profiles did not show clear clustering in response to fullerenol treatments (Fig 10A, S1 File). However, the −Fe^II^ plants were separated from those treated with fullerenol (F2). Consequently, the treatment with fullerenol of the F2 probably influenced the metabolism of the–Fe^II^ plants, although this effect is relatively weak since the differences are associated only with PC2 (16% of the variance). The OPLS-DA model included predictive and orthogonal components, R^2^X = 0.67, R^2^Y = 0.99 (p = 0.1), Q^2^Y = 0.75 (p = 0.1). A predictive component reflecting the effect of the investigated factor was associated with 24% of the variance, which is half of that for the +Fe^II^ plants. The statistical significance of the model is relatively low, which reflects weak differences between the control and the fullerenol treated plants. First, fullerenol stimulated the accumulation of amino acids and other nitrogen-containing compounds in leaves of the–Fe^II^ plants, as well as various carboxylates including oxalate, shikimate, malate, glycerate, citrate and glycolate (Fig 10B). However, some carboxylates (malonate and glutarate) were found in greater amounts in the control than in fullerenol treated plants. Also, the–Fe^II^ plants showed more sterols as compared with fullerenol treated plants. At the same time, sugars exhibited different trends in response to fullerenol addition. Results of enrichment analysis show that fullerenol stimulated the metabolism of amino acids and dicarboxylates and repressed sterol metabolism in leaves of–Fe^II^ plants (Fig 10C).

## Discussion

### Effects of Fe species and fullerenol on leaf metabolome in Fe-sufficient plants

We provide evidence that the metabolic activity of fullerenol is expressed depending Fe status of plants created either with (+Fe^III^ and +Fe^II^) or without (−Fe^III^ and −Fe^II^) Fe supply. Previously we demonstrated that the root apoplastic and total Fe contents of the +Fe^II^ cucumber plants were significantly higher as compared with the +Fe^III^ plants [22]. Ferrous (Fe^II^) species are more soluble than Fe^III^ ions. Moreover, in Strategy I plants, Fe^II^ is a substrate for trans-membrane transport [48].

In this study, metabolomics analysis revealed that ferric and ferrous ions differently influenced the metabolic pathways in the Fe-fed cucumber leaves. Whereas the Fe^II^ supply led to increased levels of primary metabolites, including various sugars, amino acids (asparagine, valine, b-alanine, threonine, isoleucine, serine, GABA and aspartate) and some organic acids (citrate and 2-ketoglutarate), the +Fe^III^ plants showed higher abundance of fatty acids and carboxylic acids, such as oxalate, pyruvate, lactate, succinate and malate (Fig 3A). The results suggest that leaf metabolomes of cucumber were re-programmed by Fe^II^ exposure towards activation pathways related mainly to sugar and amino acid metabolism, as compared with the Fe^III^ plants. In plants primary metabolites are ubiquitous and play essential roles in growth, development or reproduction. Sugars are involved in most metabolic and signalling pathways that control growth, development and stress tolerance in plants [49]. Amino acids play a crucial role in the biosynthesis of proteins and osmolytes, regulation of ion transport and many cellular enzymes [50]. Changes in the amino acid profile could indicate the re-programming of nitrogen metabolism to manage plant growth and development [51]. It has been reported that a high Fe^II^ concentration in soil solution can cause imbalances in the nutrient uptake [52]. Although leaf metabolomics pathways of Fe-fed plants were perturbed by different Fe species they, however, did not significantly alter plant dry biomass, chlorophyll (Chl) content, Chl-fluorescence parameters and elemental composition [22]. Thus, the concentration of Fe^II^ (10 μM) used in this study was probably not toxic, suggesting that metabolomics perturbations observed in the Fe^II^-fed cucumber were unlikely to be associated with toxicity of ferrous ions.

The composition of the metabolome in Fe-sufficient cucumber changed with fullerenol treatments. In the +Fe^III^ plants, fullerenol significantly activated carbohydrate metabolism, including the pentose phosphate pathway and starch metabolism, as well as histidine and ascorbate metabolism (Fig 4C). In the +Fe^II^ plants, activation by fullerenol of metabolism of lipophilic compounds, sterols and fatty acids was found; on the other hand, fullerenol induced repression of carbohydrate metabolism as well as of exchange of amino acids (Fig 5C). The up-regulation of the pentose phosphate pathway in Fe^III^-fed plants indicates that fullerenol perturbed energy associated pathways, suggesting the positive effects on plants with lower levels of active Fe as compared with Fe^II^-fed plants. Thus, fullerenol was effectively involved in the metabolic processes of the Fe-sufficient cucumber plants. Despite the perturbation in cucumber metabolism, growth and other physiological indexes, such as Chl-fluorescence, Chl and Fe contents remained unchanged by fullerenol, at least for 10 days, indicating that fullerenol was not harmful to cucumber plants at the concentrations used [22].

### Effects of Fe species and fullerenol on leaf metabolome in Fe-deficient plants

Iron starvation dramatically affects plant growth and metabolism. Cucumber plants grown in Fe-free solutions were chlorotic, especially the −Fe^III^ than–Fe^II^ plants. Being higher in root apoplastic Fe, the–Fe^II^ plants became less chlorotic and were more tolerant of Fe deficiency, as compared with the–Fe^III^ plants [22]. Plants have evolved adaptive metabolic responses to maintain homeostasis under adverse environmental conditions. Metabolic consequences of stress induced by temperature, water and salinity, sulfur, phosphorus, oxidative and heavy metals have often been discussed [23,24,53–55]. Generally, the plant defense responses are associated with the production of at least three different types of compounds: (1) antioxidants or osmoprotectants; (2) products of the stress metabolism; and (3) signaling molecules involved in mediating metabolic responses [53]. Various environmental stresses induce the accumulation of small molecules with antioxidative activity, such as ascorbic acid, amino acids (proline), sugars, ascorbic acid, glutathione, α-tocopherols, carotenoids and flavonoids [54]. As a whole, plant metabolic responses depend highly on the type of stress (biotic or abiotic), tissues, species and plant-pathogen or pest interactions [56]. The diversity of stress-responsive metabolites that accumulate in plants indicates that various pathways can be activated that respond to specific environmental insults.

To sustain the increased Fe uptake in Fe-deficient plants, several changes occur at the metabolic level. These changes include accumulation of carbohydrates, amino acids and especially organic acids, primarily malate and citrate; increases in activity of several enzymes of the Krebs cycle and the glycolytic pathway [7,57–59]. In Strategy I Fe-starved plants, phosphoenolpyruvate carboxylase (PEPC) is one of the most important metabolic activities [60]. This enzyme is involved in the fixation of bicarbonate to phosphoenolpyruvate (PEP) to produce oxaloacetate (OAA) and inorganic phosphorus. Activation of PEPC under Fe deficiency is thought a driving force for glycolysis that leads to an increase in the production of cytosolic acidification to activate H^+^-ATPase [57]. In addition, PEPC could be responsible for the production of organic acids, which facilitate the acquisition of Fe and its transport in plants [61].

In this research, we detected a large number of specific metabolic alterations in cucumber leaves triggered by different Fe species. Indeed, the more suffering −Fe^III^ plants had higher abundances of various sugars (sucrose), amino acids (proline, alanine, GABA, oxoproline and aspartate) and carboxylic acids (isopropyl malate, malonate, citramalate, malate, citrate, malate and fumarate), than the −Fe^II^ plants. By contrast, the −Fe^II^ plants exhibited higher abundances in lipophilic compounds (fatty acids, acylglycerols and sterols) and some carboxylates (oxalate, glycolate, succinate, lactate) (Fig 3). More specifically, whereas a lack of Fe^III^ activated metabolism of aliphatic amino acids and repressed lipid metabolism, a lack of Fe^II^ activated lipid metabolism and repressed the metabolism of amino acids, sugars, including starch metabolism and carbon fixation (Fig 7). The largest metabolite groups played important role in metabolite correlation were “lipophilic” and “carbohydrate-amino acid” (Fig 8). Whereas Fe^III^ deficiency reduced the number of strong correlations of metabolite-metabolite in comparison to the +Fe^III^ plants, Fe^II^ deficiency induced opposite changes (Fig 8), suggesting that a lack of Fe^II^ caused larger scale metabolic rearrangements in comparison with omitting Fe^III^.

The up-regulation of sugars, amino acids and organic acids observed in the–Fe^III^ plants is consistent with several studies that analysed Fe deficiency and other abiotic responses of plants. Sugar metabolism plays significant roles in Fe deficiency. High concentrations of metabolites such as glucose and sucrose are of importance (i) for the transport and storage of carbon in abiotic stresses acting as compatible solutions for protection, (ii) as an energy source in plant metabolism and (iii) as signalling molecules [62,63]. Levels of sucrose were increased in most stress conditions [64]. A large increase in sucrose and fructose was found in Fe stressed leaves of *Malus halliana* [63]. In *Arabidopsis*, increased sucrose accumulation can regulate Fe deficiency responses by promoting auxin signalling [65]. Increases in the abundance of few amino acids were found in the Fe-stressed roots of *Prunes* rootstocks and *Arabidopsis thaliana* [59,66]. The accumulation of amino acids and derivatives has been related to plant responses and adaptation to various stresses [64], including Mg deficiency [32]. It has been suggested that Fe deficiency can activate N cycling and protein catabolism in roots of *Medicago truncatula* [67]. The amino acid proline is known as a compatible solute in plants and accumulated under various stress conditions [64]. GABA is another metabolite largely and rapidly produced in response to stresses, which protect plants due to regulation of cytosolic pH, protection against oxidative stress and functions as an osmoregulator and a signalling molecule [64]. However, in cucumber roots, a decrease in the abundance of the majority amino acids was observed under Fe deficit [68]. Zinc (Zn) deficiency can also cause decrease carbohydrate and nitrogenous metabolites in the tea leaves [33].

Several authors have suggested that Fe deficiency induces the activation of the tricarboxylic acid (TCA) cycle, which provides abundant organic acids and proton for the acidification of the rhizosphere. It has been reported that Fe deficiency caused an increase in the organic acid pool, especially citrate and malate [7,60,61]. Many organic compounds have been proposed as strong cation chelators, which can play an important role in facilitating nutrient uptake, including the mobilisation of Fe in root apoplast [1,69]. Organic acids played key role in the regulation of metabolism in Zn-stressed tea leaves [33]. It has been reported that citrate is the most likely candidate for Fe xylem transport [70]. Additionally, citrate plays a role in the regulation of cellular pH and as a carbon skeleton source [60]. Various organic acids including oxaloacetate, succinate, fumarate and malate, as TCA cycle intermediates are the precursors for various biosynthetic pathways, such as gluconeogenesis and amino acid synthesis [71]. Taken together these observations suggest a crucial role for the Fe valence state of Fe sources in cucumber metabolic responses to Fe deficiency.

Our results showed that fullerenol treatment altered the physiological and metabolic responses of cucumber plants to Fe deficiency in a Fe species-dependent manner. Our previous physiological study demonstrated that fullerenol significantly lowered the root apoplastic Fe in the–Fe^II^-starved plants, in turn, increasing the leaf active-Fe concentration and successful suppression of plant Fe deficiency symptoms. At the same time, the beneficial effects of fullerenol were not obvious in the Fe^III^-starved plants [22]. In the current study, several changes occurred at the metabolic level in response to fullerenol. In the–Fe^III^ plants, metabolites changed by fullerenol differently. The level of some carboxylates, such as adipate, ferulate, levulinate, glutarate and succinate, increased, whereas the level of other carboxylates, such as fumarate, malonate, malate and citrate, decreased (Fig 9B). In addition, amino acid abundance decreased in cucumber leaves after fullerenol treatments of–Fe^III^ plants. Contrastingly, in leaves of the–Fe^II^ plants fullerenol increased the abundance of amino acids and other nitrogen-containing compounds, as well as carboxylates, such as oxalate, shikimate, malate, glycerate, citrate and glycolate (Fig 10B). Likewise, fullerenol-nanopriming promoted primary metabolism to enhance growth and productivity of spring wheat under salinity stress [18]. Generally, in the leaves of the–Fe^II^ plants, fullerenol stimulated the metabolism of amino acids and dicarboxylates and repressed sterol metabolism (Fig 10C). These metabolic alterations induced by fullerenol in Fe-stressed plants seem in agreement with the previously reported physiological responses of these plants. Indeed, the triggering by fullerenol of up-regulation of carboxylates, primarily citrate and malate, in the–Fe^II^ plants may be a Fe species-dependent advantage, which could contribute to correct mobility of Fe throughout the cucumber plants. Other metabolic changes lack obvious connections to Fe homeostasis; however, they could become informative in future studies.

### Underlying mechanisms of fullerenol impact on leaf metabolome

The underlying mechanisms of carbon-based nanomaterials interactions in various plant systems are less understood than that of metallic nanoparticles widely used by most of the plant technology researches [26,35]. Therefore, the mechanisms by which fullerenol changes plant metabolomics remain unclear. However, fullerenol has potential as a plant growth regulator. Many studies have suggested that fullerenol is mobile in plant tissues and can penetrate through different biomembranes [20,72]. Small particles of fullerenol have been found in the root vascular cylinder of wheat [73]. It has been reported that in wheat, ^13^C-fullerenol was transported from roots to the stems and leaves [73]. Moreover, in wheat leaves, fullerenol increased chlorophyll content. Small size and good solubility allow fullerenol [C_60_(OH)_20_] to readily permeate through the cell wall of plants (*Allium cepa*), likely due to a concentration gradient [74]. Being hydrophilic, fullerenol is largely excluded by the plasma membrane and accumulated between the cell wall and the plasma membrane of plant cells [74]. At high concentrations, fullerenol can induce a loss of membrane integrity that can affect membrane transport of nutrients [74]. In this study, the Fe-status was a leading factor influenced cucumber metabolome (Fig 2), suggesting that fullerenol may alter the metabolomics responses of plants through the alteration of Fe availability, for example in the–Fe^II^ plants [22]. Indeed, it is known that in the fullerenol-Fe^III^ complex, fullerenol can directly reduce Fe^III^ to Fe^II^ via electron transfer [75]. Fullerenol is known to be rich in OH groups. Therefore, positive ferrous ions can bind with negatively charged nanoparticles of fullerenol and shift fullerenol surface charge to the more positive values, thereby creating a delivery system for Fe^II^ [76]. Further, seed priming with fullerenol increased K^+^ uptake and decreased Na^+^/K^+^ ratio in shoot of salinity stressed wheat that can be helpful for alleviation ion toxicity at competitive growth conditions [18]. Taken together, these results suggest that fullerenol might improve tissue ion homeostasis in stressed plants.

In this study, Fe deficiency conditions were created with the exception of available Fe from a nutrient solution. As a result, the hydroponically grown cucumber plants exhibited the Fe deficiency symptoms typical for soil-grown Strategy I plants: restricted growth, leaf chlorosis, enhanced FC-R activity and proton extrusion by roots. It seems therefore, the conclusions from this study can be applicable for Fe-deficient soil-grown plants. However, further studies are necessary to elucidate the mechanisms involved in fullerenol-mediated metabolomics responses in higher plants, including soil-grown plants.

## Conclusions

Fullerenol was effectively involved in the metabolic processes of cucumber plants and it was not harmful at the used concentrations. Our results showed for the first time the leading role of Fe-status of plants in their metabolomics responses to fullerenol treatments. Whereas in the Fe^III^-fed plants, fullerenol activated metabolisms of carbohydrates and amino acids, in the Fe^II^-fed plants fullerenol activated metabolism of lipophilic compounds and repressed metabolism of carbohydrates and amino acids. The fullerenol-induced acceleration of the pentose phosphate pathway in Fe^III^-fed plants suggests that fullerenol can perturbed energy associated pathways in plants with lower levels of active Fe as compared with Fe^II^-fed plants. In the Fe^III^-deficient plants, fullerenol stimulated metabolism of C_3_ carboxylates and lipophilic compounds with repressing metabolism of amino acids, hexoses and dicarboxylates. At the same time, in the Fe^II^-deficient plants, fullerenol stimulated the metabolism of amino acids and dicarboxylates and repressed sterol metabolism. The metabolic perturbations in the Fe^III^-treated plants induced by fullerenol were the opposite kind from those in the Fe^II^-treated plants, suggesting that the state of Fe sources is of importance for re-programming of cucumber metabolome responses to fullerenol. This study not only shows new insights into the fullerenol induced metabolic perturbation but also provides important data on metabolites connecting with Fe homeostasis for future investigations.

## Supporting information

S1 Table Compound identification in the cucumber leaves grown hydroponically in a nutrient solution, either with (+Fe^II^ and +Fe^III^) or in Fe-free (−Fe^II^ and −Fe^III^) nutrient solution, with or without the supply of 0 (F0), 1 (F1) and 2 (F2) mg L^-1^ fullerenol for 10 days. (XLSX)

S2 Table Metabolite content in the cucumber leaves grown hydroponically in a nutrient solution, either with (+Fe^II^ and +Fe^III^) or in Fe-free (−Fe^II^ and −Fe^III^) nutrient solution, with or without the supply of 0 (F0), 1 (F1) and 2 (F2) mg L^-1^ fullerenol for 10 days. (XLSX)

S1 File Metabolite responses in the cucumber leaves. (DOCX)
