# Supplementary material for: Fullerenol changes metabolite responses differently depending on the iron status of cucumber plants
Source: PLoS One. 2021 May 17;16(5):e0251396. doi: 10.1371/journal.pone.0251396 (PMC8128279; doi:10.1371/journal.pone.0251396)

**S1 Fig. Histograms of frequencies of Pearson correlation coefficients (r) of metabolite arbitrary content in the cucumber leaves grown hydroponically in a nutrient solution, either with (+Fe^II^ and +Fe^III^) or in Fe-free (−Fe^II^ and −Fe^III^) nutrient solution, with or without the supply of 0 (F0), 1 (F1) and 2 (F2) mg L^-1^ fullerenol for 10 days.**
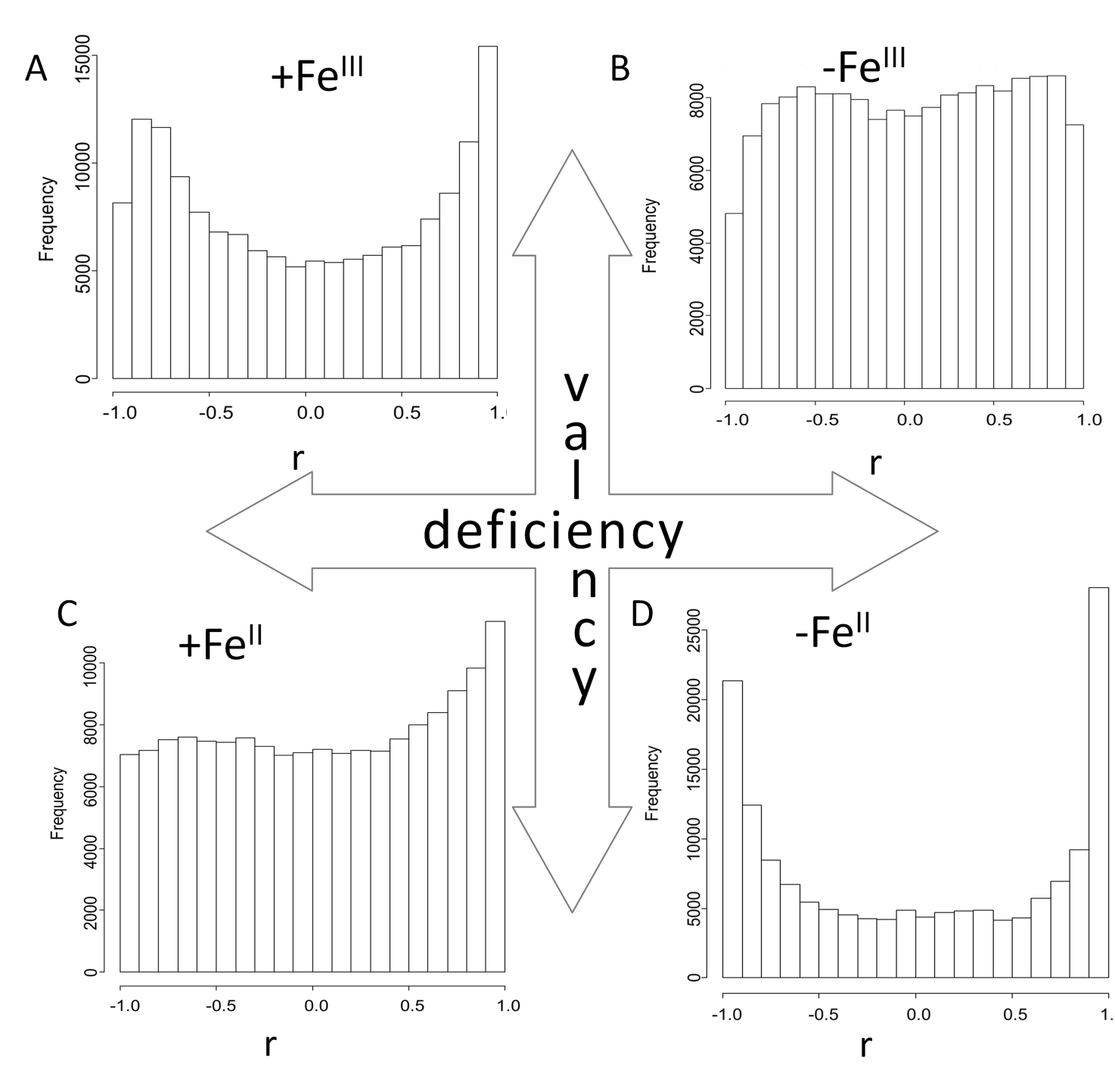


**S2 Fig. Effects of fullerenol on leaf metabolites of cucumber plants in response to removing Fe^III^ from a nutrition solution for 10 days.**

A. Factor loadings of predictive components (VIP >1) from OPLS-DA model (R^2^X = 0.74, R^2^Y = 0.99 (*p* = 0.02), Q^2^Y = 0.97) (*p* = 0.02) comparing the Fe^III^-sufficient and Fe^III^-deficient plants treated with fullerenol (F1) (1 mg L^-1^). Positive loadings correspond to higher metabolite contents under +Fe^III^ conditions.

В and C. SUS-plots: the scattering of metabolites in the space of factor loadings from OPLS-DA models for comparing the Fe^III^-sufficient and Fe^III^-deficient plants grown with or without the supply of 0 (F0), 1 (F1) and 2 (F2) mg L^-1^ fullerenol.

D. SUS-plot: the scattering of metabolites in the space of factor loadings from the OPLS-DA model for comparing the Fe^III^-sufficient and Fe^III^-deficient plants treated with F1 and OPLS-DA model for comparing Fe^III^-sufficient and Fe^III^-deficient untreated plants.


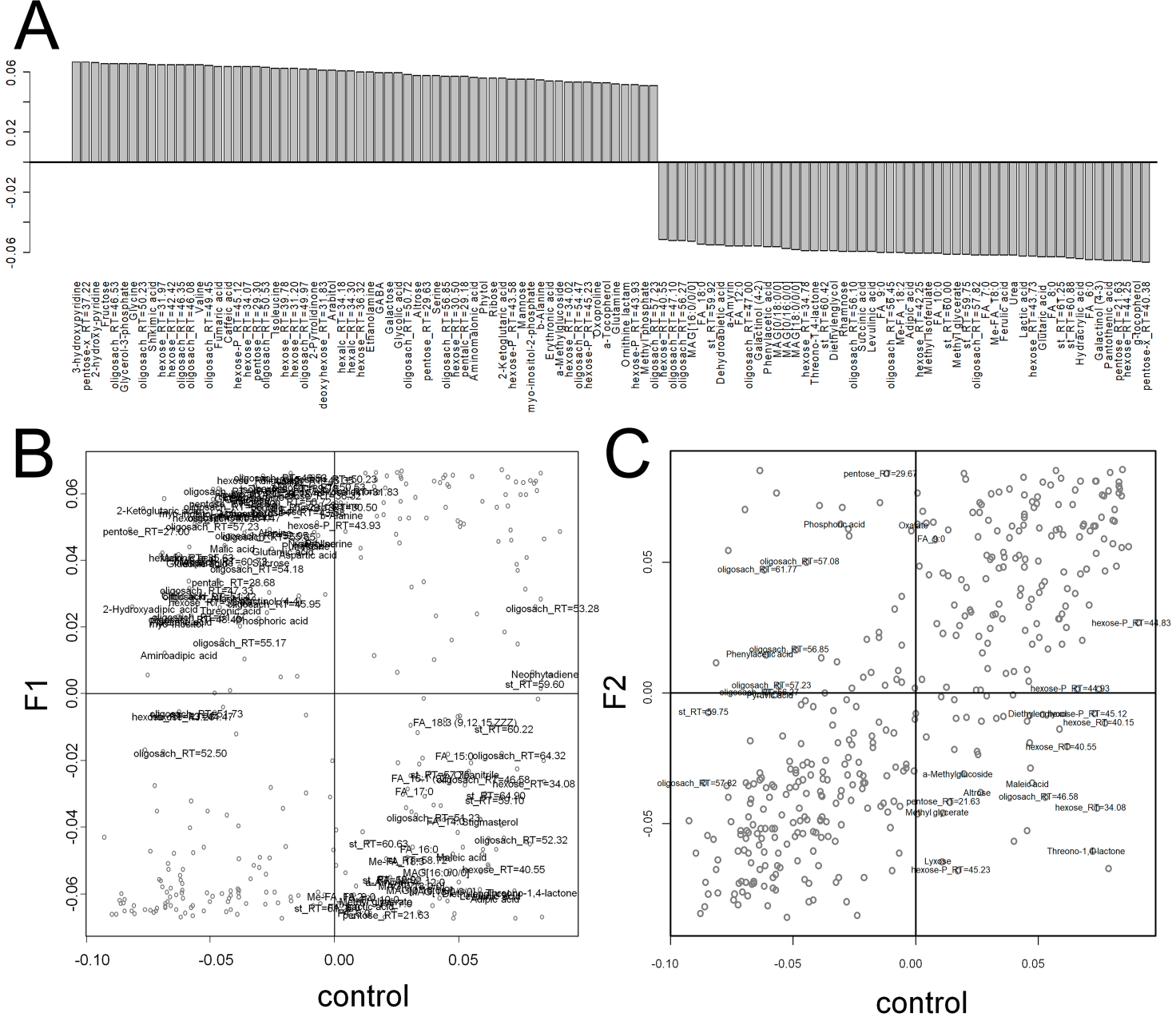


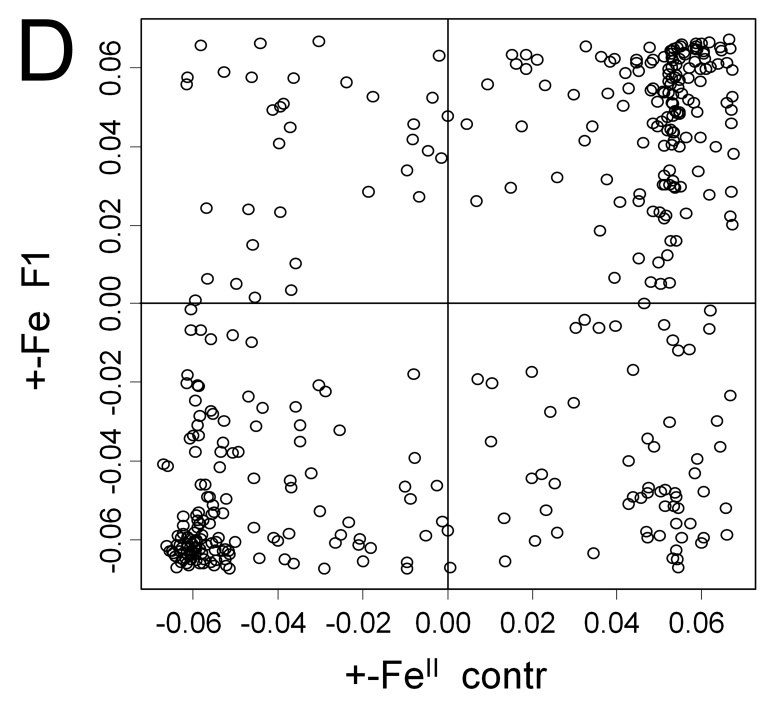


**S3 Fig. Effects of fullerenol on leaf metabolites of cucumber plants in response to removing Fe^II^ from a nutrition solution for 10 days.**

SUS-plots: the scattering of metabolites in the space of factor loadings from OPLS-DA models for comparing the Fe^II^ -sufficient and Fe^II^ -deficient plants grown with or without the supply of 0 (F0), 1 (F1) and 2 (F2) mg L^-1^ fullerenol.


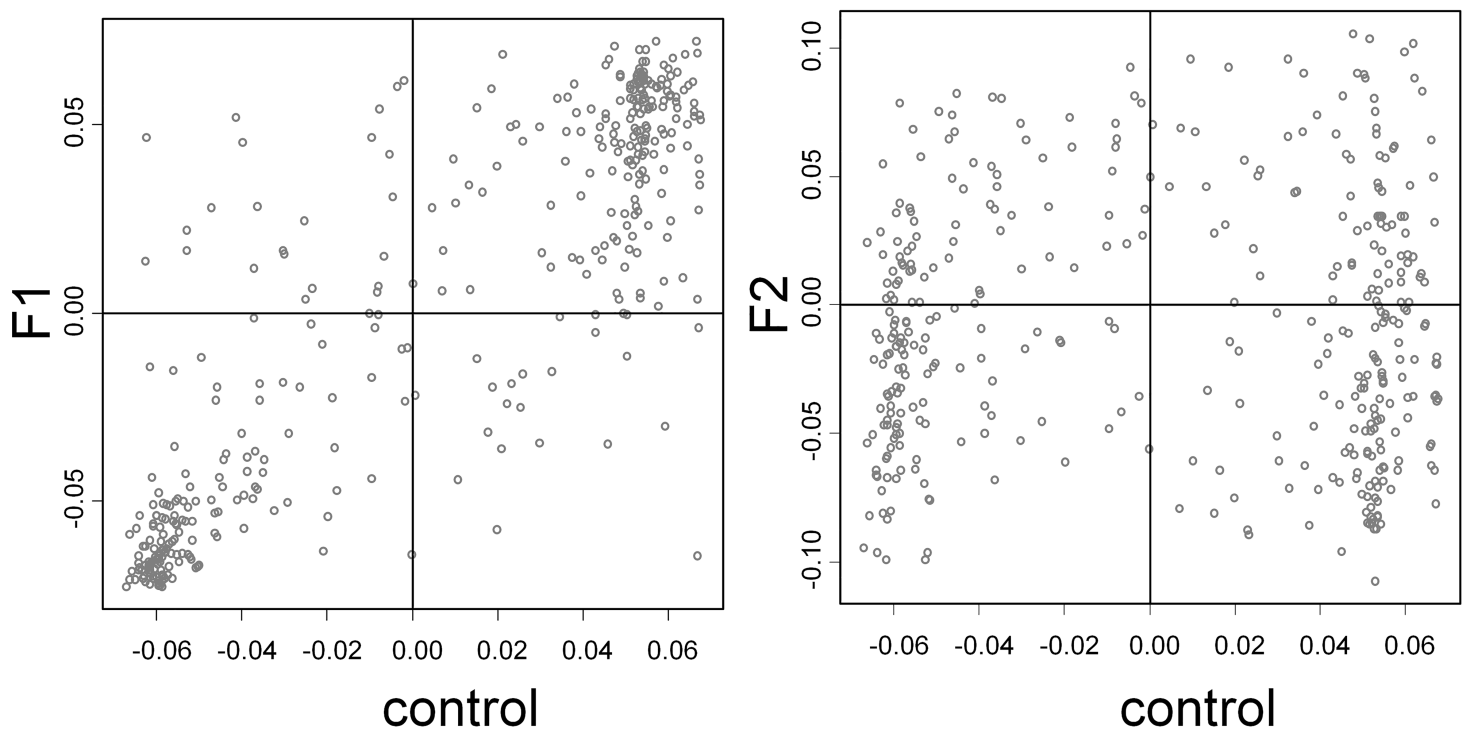

Supplement: S1 File — (DOCX) [file pone.0251396.s003.docx]
